# Correction for Snoeyenbos-West et al., “Cultivating efficiency: high-throughput growth analysis of anaerobic bacteria in compact microplate readers”

**DOI:** 10.1128/spectrum.03322-24

**Published:** 2025-02-24

**Authors:** Oona L. O. Snoeyenbos-West, Christina R. Guerrero, Makaela Valencia, Paul Carini

## AUTHOR CORRECTION

Volume 12, no.5, e03650-23, 2024, https://doi.org/10.1128/spectrum.03650-23. Page 2: Lines 6–11 should read as follows: “Measurement of OD from cultures in these types of tubes is labor-intensive, low throughput, and relies heavily on the efficiency of the researcher conducting the experiments, who must manually pierce the rubber septa with a needle to extract a small volume of culture to be read by a spectrophotometer. Recently, a new tool capable of multiplexing biological replicates in real time using standard laboratory glassware, has automated this procedure (14).”

