# Supplementary material for: Cultivating efficiency: high-throughput growth analysis of anaerobic bacteria in compact microplate readers
Source: Microbiol Spectr. 2024 Mar 19;12(5):e03650-23. doi: 10.1128/spectrum.03650-23 (PMC11064495; doi:10.1128/spectrum.03650-23)
Supplement: Figures S1 and S2 — Growth curves of E. coli incubated in aerobic and anaerobic conditions. [file spectrum.03650-23-s0001.docx]

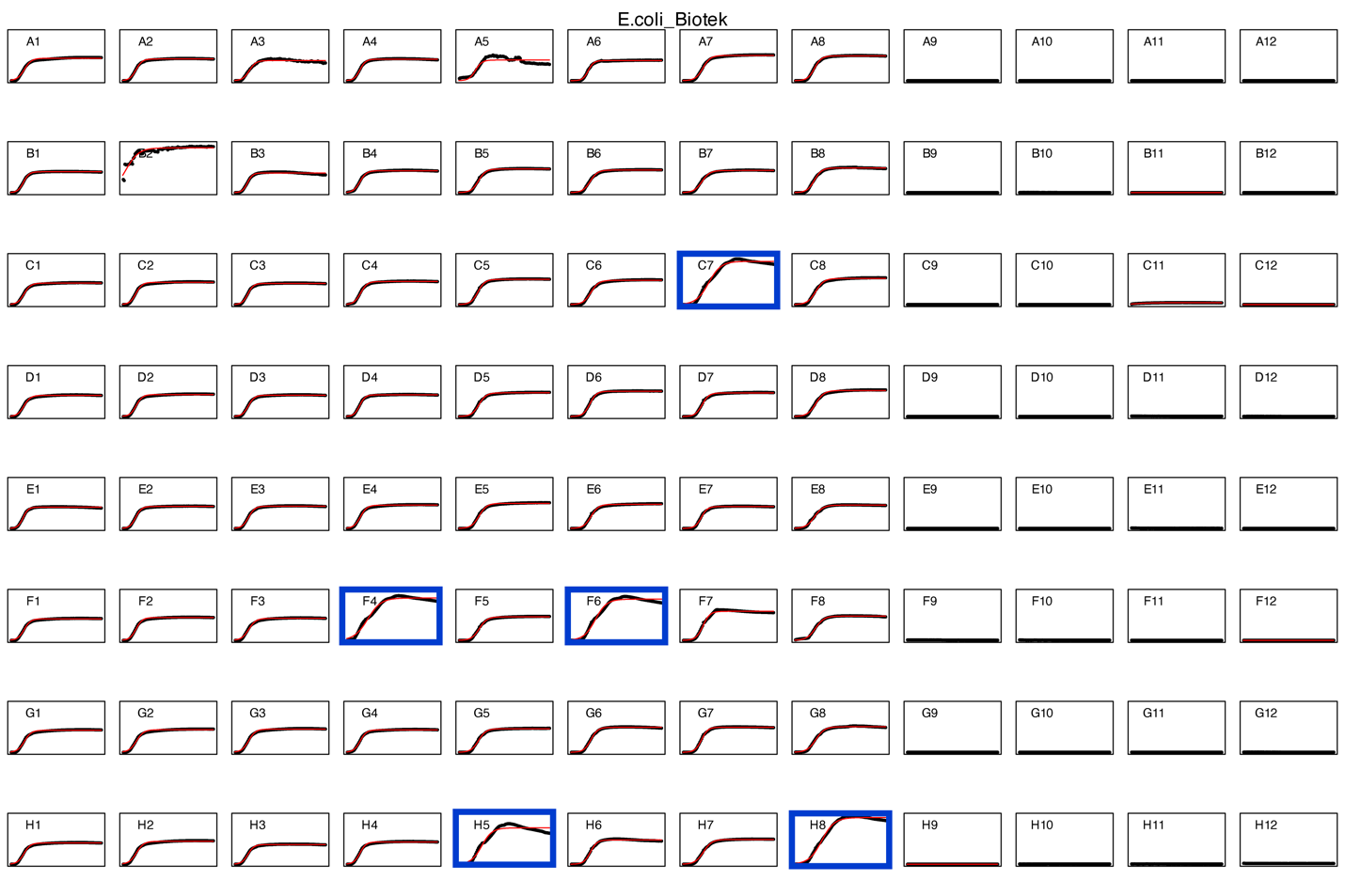


Supplementary Figure 1: Growth curves of *E. coli* incubated in aerobic conditions in a BioTek Synergy HTX microplate reader. Columns 1-8 were inoculated with cells. Columns 9-12 were uninoculated media blanks. The black lines are plotted from OD600 measurements collected by the microplate reader. The overlaying red lines are best-fit curves modeled from Growthcurver. Curves inside of blue boxes are growth curve variants that had a high carrying capacity and a lower growth rate, leading to a higher AUC, relative to the remaining wells.


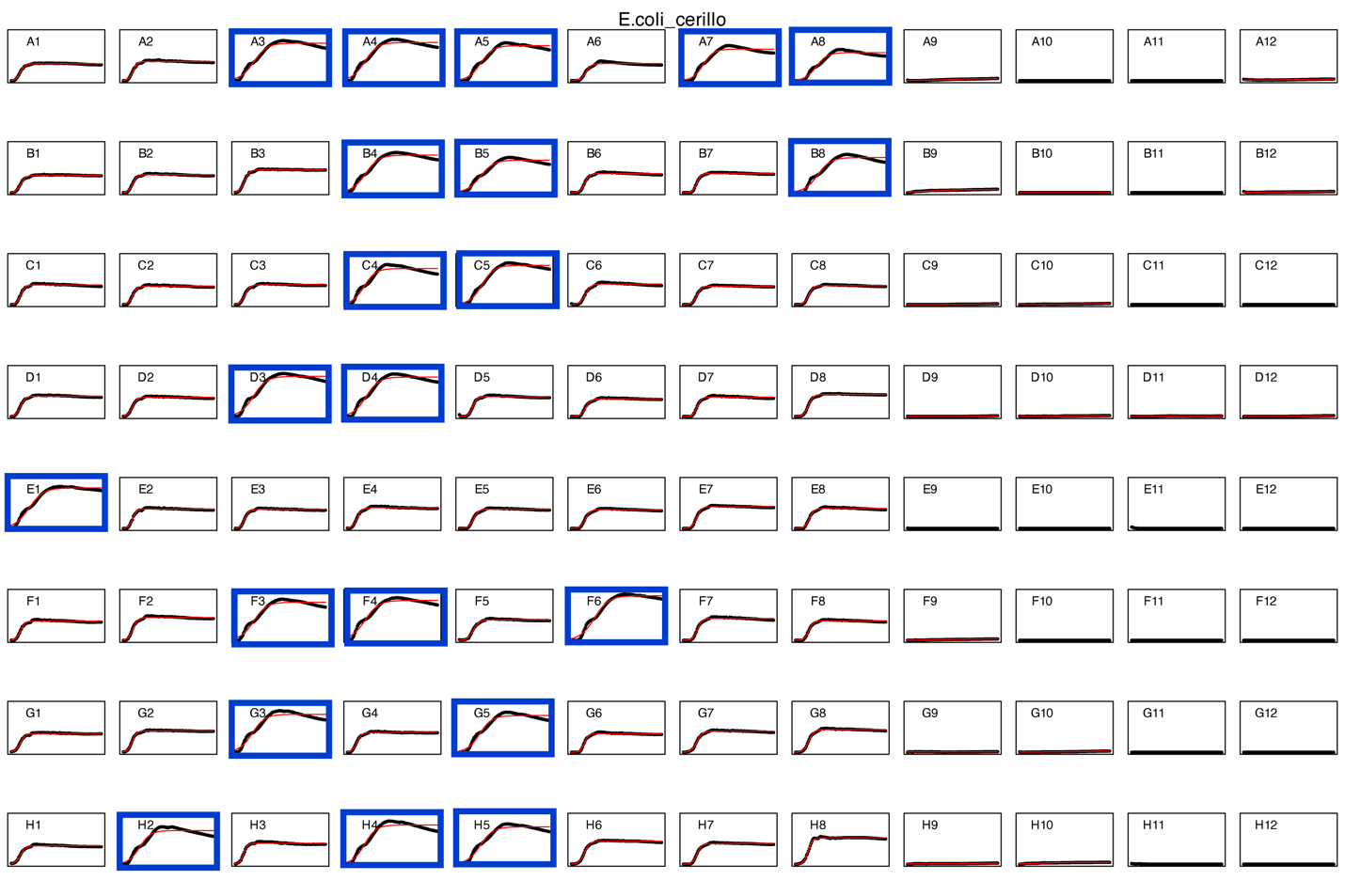


Supplementary Figure 2: Growth curves of *E. coli* incubated in aerobic conditions in a Cerillo Stratus microplate reader. Columns 1-8 were inoculated with cells. Columns 9-12 were uninoculated media blanks. The black lines are plotted from OD600 measurements collected by the microplate reader. The overlaying red lines are best-fit curves modeled from Growthcurver. Curves inside of blue boxes are growth curve variants that had a high carrying capacity and a lower growth rate, leading to a higher AUC, relative to the remaining wells.
